# Molecular phylogeny inferred from the mitochondrial genomes of Plecoptera with *Oyamia nigribasis* (Plecoptera: Perlidae)

**DOI:** 10.1038/s41598-020-78082-y

**Published:** 2020-12-01

**Authors:** Meng-Yuan Zhao, Qing-Bo Huo, Yu-Zhou Du

**Affiliations:** 1 School of Horticulture and Plant Protection & Institute of Applied Entomology, Yangzhou University, Yangzhou, 225009 China; 2 Joint International Research Laboratory of Agriculture and Agri-Product Safety, the Ministry of Education, Yangzhou University, Yangzhou, 225009 China

**Keywords:** Developmental biology, Evolution, Genetics, Molecular biology, Systems biology

## Abstract

In this study, the mitochondrial genome of the stonefly, *Oyamia nigribasis* Banks, 1920 (Plecoptera: Perlidae), was sequenced and compared with the mtDNA genomes of 38 other stoneflies and two Ephemerae. The *O. nigribasis* mitogenome is a circular 15,923 bp molecule that encodes a large, noncoding control region (CR) and 37 typical mtDNA genes; these include 13 protein-coding genes (PCGs), 22 transfer RNA genes (tRNAs), and two ribosomal RNA genes (rRNAs), respectively. Most of the PCGs initiated with ATN and terminated with TAN. The dihydrouridine (DHU) arm of tRNA^*Ser* (*AGN*)^ was missing, whereas the other 21 tRNAs all exhibited the typical cloverleaf secondary structure. Stem-loop (SL) structures and tandem repeats were identified in the CR. Phylogenetic analyses using Bayesian inference and maximum likelihood were undertaken to determine relationships between stoneflies. Results indicated that the Antarctoperlaria, which contains Gripopterygidae, was absolutely separated from Arctoperlaria; this finding agrees with morphology. Finally, the overall relationships could be summarized as follows ((((Notonemouridae + Nemouridae) + Leuctridae) + (Scopuridae + (Capniidae + Taeniopterygidae))) + (((Perlodidae + Chloroperlidae) + Perlidae) + (Pteronarcyidae + (Peltoperlidae + Styloperlidae))) + ((Diamphipnoidae + Eustheniidae) + Gripopterygidae)).

## Introduction

The Plecoptera order of stoneflies is a basal infraorder of the Neoptera; it is dispersed worldwide (except Antarctica) and contains ancient hemimetabolous insects^1^. Stonefly larvae frequently inhabit clean rivers and streams and are quite sensitive to dirty, polluted environments; thus stoneflies are an important bioindicator of water quality^2–4^.

The typical metazoan mitochondrial genome includes a noncoding sequence called the control region (CR) and 37 genes including 13 protein coding genes (PCGs), 22 tRNAs, two rRNAs^5,6^. The analysis of mitochondrial genomes has profoundly influenced the genetics and taxonomy of insects^7–12^. The development of whole genome sequencing technologies for insects has been slow in contrast to mtDNA; however, mitochondrial barcodes and sequences are commonly used for insect species identification^13–16^. The mtDNA sequences of representative species in all Insecta orders have been deposited in GenBank and exceed 1,500 entries^17^.

Despite previous studies, conclusions on the phylogeny and biogeography of stoneflies are inconsistent, especially concerning the family composition in the highest systematic categories^18–23^. There is some controversy on the phylogeny of Plecoptera; thus, we obtained the mtDNA sequence of *Oyamia nigribasis* and constructed phylogenetic trees based on PCG sequences to deduce the phylogenetic relationships of 39 stonefly species.

## Results and discussion

### Genome annotation and base composition

**Figure 1:**
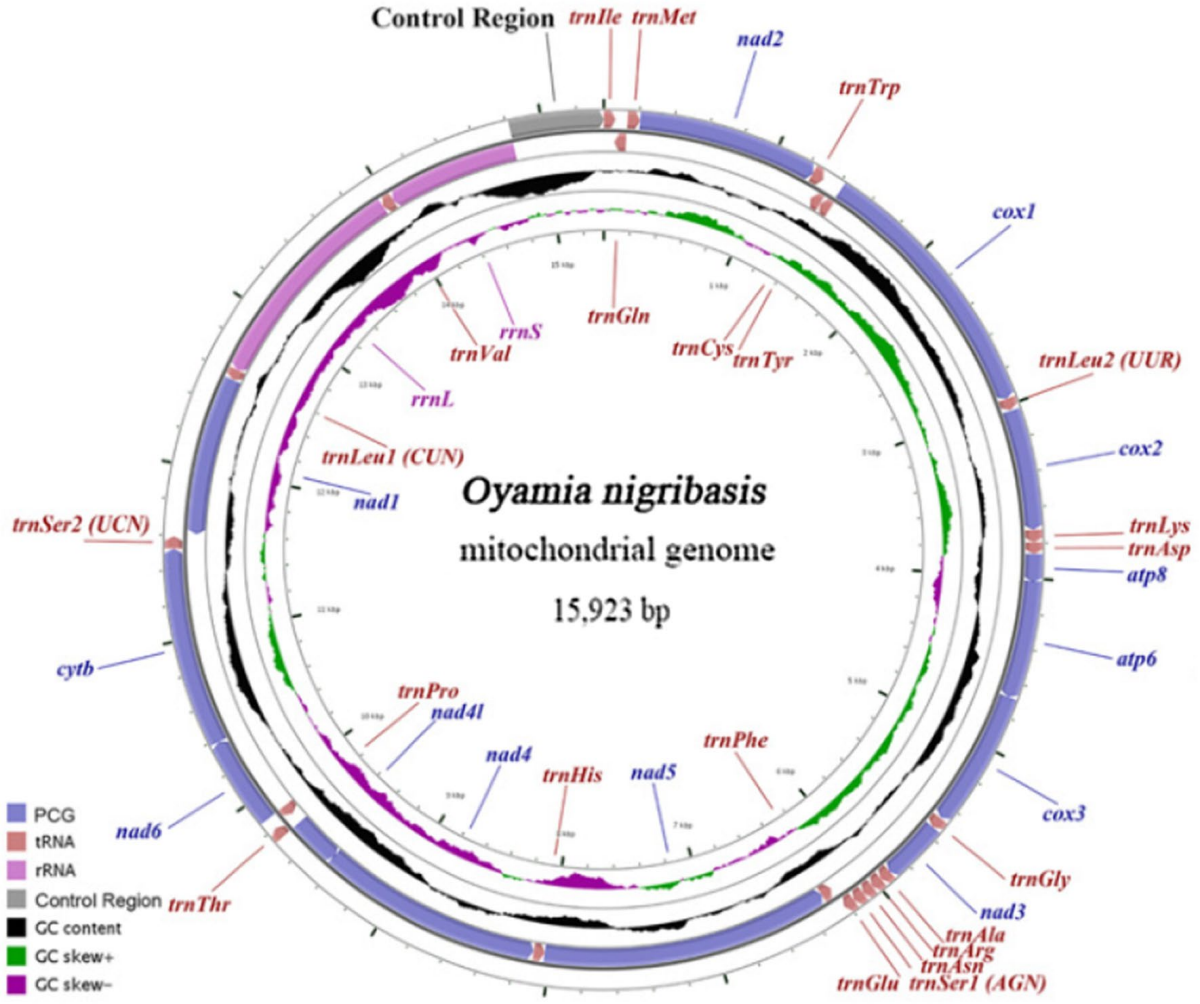
Mitochondrial map of *Oyamia nigribasis*. Genes outside the map are transcribed clockwise, while genes inside are transcribed counterclockwise. The interior circles show GC content and GC skew, which are plotted as the deviation from the average value of the entire sequence.

The *O. nigribasis* mitogenome is a circular 15,923 bp molecule, and contains the typical set of 37 mtDNA genes (13 PCGs, 22 tRNAs, and two rRNAs) along with a noncoding control region (CR) of 1022 bp. Among the 37 genes, nine PCGs and 14 tRNAs were majority strand (J-strand); four PCGs, eight tRNAs, and two rRNAs were minority strand (N-strand) (Fig. 1, Table 1). The gene arrangement in *O. nigribasis* mtDNA was highly conserved with other sequenced stoneflies and identical to the mitogenome of *Drosophila yakuba*, which is regarded as the putative ancestral arthropod^24^. The mitogenome of *O. nigribasis* contains 11 pairs of adjacent overlapping genes covering 41 nucleotides; 10 pairs were unlinked and encompassed 48 intergenic nucleotides (IGNs). The shortest overlap was 1 bp (multiple sites), whereas the longest was 9 bp and mapped between *trnTyr (Y)* and *cox1* (Table 1). The shortest interval between genes was 1 bp (two sites) while the longest was a 16-bp intergenic region between *trnSer2 (UCN)* and *nad1* (Table 1).

**Table 1:** Annotation of the *Oyamia nigribasis* mitogenome.

Gene	Position (bp)	Size (bp)	Direction	Intergenic Nucleotides (IGN)	Anti- or Start / Stop Codons	A + T%
*trnIle(I)*	1–66	66	Forward	0	GAT	66.6
*trnGln(Q)*	64–132	69	Reverse	-3	TTG	69.6
*trnMet(M)*	137–205	69	Forward	4	CAT	63.8
*nad2*	206–1240	1035	Forward	0	GTG/TAA	70.7
*trnTrp(W)*	1239–1306	68	Forward	-2	TCA	69.2
*trnCys(C)*	1299–1364	66	Reverse	-8	GCA	63.6
*trnTyr(Y)*	1364–1432	69	Reverse	-1	GTA	64.7
*cox1*	1424–2963	1540	Forward	-9	ATC/T-	64.0
*trnLeu2(UUR)*	2964–3029	66	Forward	0	TAA	69.6
*cox2*	3038–3725	688	Forward	8	ATG/T-	64.5
*trnLys(K)*	3726–3796	71	Forward	0	CTT	66.2
*trnAsp(D)*	3796–3863	68	Forward	-1	GTC	80.9
*atp8*	3864–4025	162	Forward	0	ATT/TAA	69.7
*atp6*	4019–4696	678	Forward	-7	ATG/TAA	68.9
*cox3*	4700–5488	789	Forward	3	ATG/TAA	62.4
*trnGly(G)*	5488–5556	69	Forward	0	TCC	75.4
*nad3*	5557–5910	354	Forward	0	ATT/TAA	74.6
*trnAla(A)*	5914–5979	66	Forward	3	TGC	74.2
*trnArg(R)*	5979–6043	65	Forward	-1	TCG	63.1
*trnAsn(N)*	6043–6109	67	Forward	-1	GTT	71.6
*trnSer1(AGN)*	6110–6176	67	Forward	0	GCT	62.7
*trnGlu(E)*	6177–6243	67	Forward	0	TTC	88.1
*trnPhe(F)*	6245–6312	68	Reverse	1	GAA	69.1
*nad5*	6313–8047	1735	Reverse	0	ATG/T-	71.0
*trnHis(H)*	8048–8116	69	Reverse	0	GTG	66.7
*nad4*	8117–9457	1341	Reverse	0	ATG/TAA	71.9
*nad4l*	9451–9747	297	Reverse	-7	ATG/TAA	74.4
*trnThr(T)*	9750–9817	68	Forward	2	TGT	77.9
*trnPro(P)*	9819–9884	66	Reverse	0	TGG	71.2
*nad6*	9886–10,407	522	Forward	1	ATT/TAA	71.7
*Cytb*	10,407–11,543	1137	Forward	-1	ATC/TAG	64.8
*trnSer2(UCN)*	11,542–11,609	68	Forward	0	TGA	77.9
*nad1*	11,626–12,576	951	Reverse	16	ATG/TAG	69.6
*trnLeu1(CUN)*	12,579–12,646	68	Reverse	2	TAG	66.2
*rrnL*	12,647–14,010	1362	Reverse	0		72.8
*trnVal(V)*	14,011–14,082	72	Reverse	8	TAC	66.7
*rrnS*	14,083–14,901	819	Reverse	0		70.2
CR	14,902–15,923	1022		0		72.7

Similar A + T contents were observed for the entire *O. nigribasis* mtDNA molecule, PCGs, tRNAs, rRNAs, and CR, which were 70.2%, 69.1%, 70.2%, 71.5%, and 72.7%, respectively (Table 2). The lowest and highest A + T content was 62.4% for *cox3* and 88.1% for *trnGlu (E)*, respectively (Table 1). The AT- and GC-skew expressed positively and negatively, respectively, which is consistent with other stonefly mitogenomes (Table 2).

**Table 2:** The composition of nucleotides in different regions of *Oyamia nigribasis*.

Species	Whole mtDNA genome				PCGs		tRNAs		rRNAs		Control region	
	Size (bp)	A + T (%)	AT-skew	GC-skew	Size (bp)	A + T (%)	Size (bp)	A + T (%)	Size (bp)	A + T (%)	Size (bp)	A + T (%)
*Oyamia nigribasis*	15,923	70.2	+	–	11,229	69.1	1426	70.2	2181	71.5	1022	72.7

### Protein-coding genes

**Figure 2:**
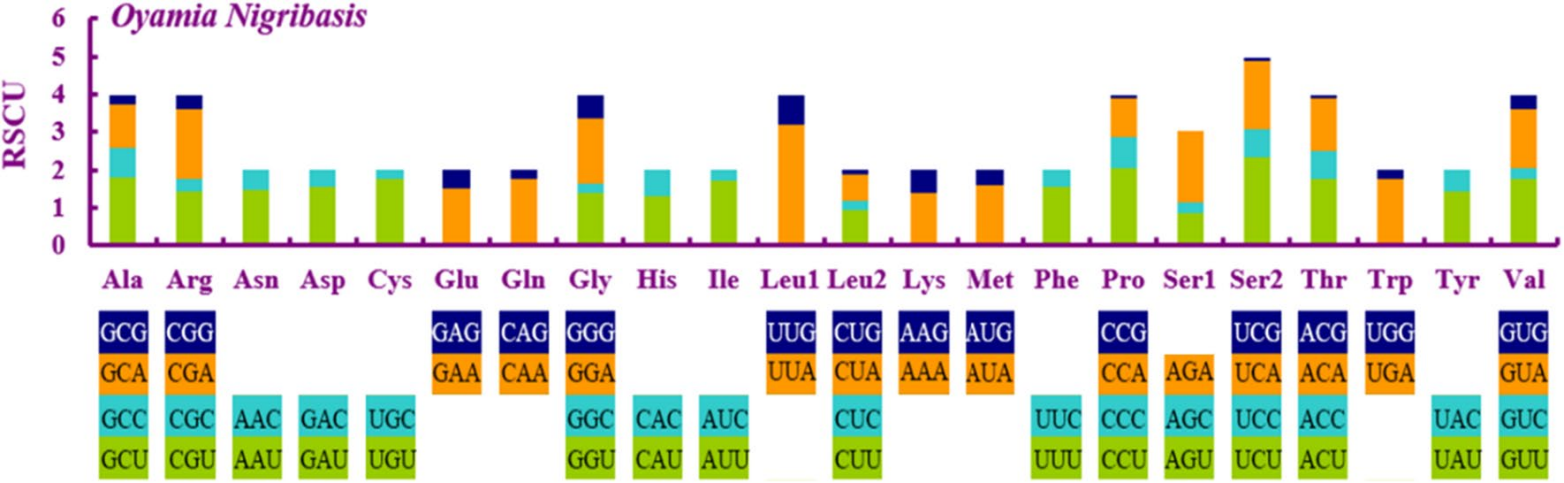
Relative synonymous codon usage (RSCU) in *O. nigribasis*.

*O. nigribasis* PCG were similar in size (65–71 bp), whereas the A + T content varied from 62.7–88.1% (Tables 1, 2). The majority of PCGs possessed the standard start codon ATN (ATT, ATC or ATG); however, *nad2* started with GTG, which has also previously been observed for other stonefly such as *Taeniopteryx ugola*^10^. Furthermore, most PCGs terminated with complete codons (e.g. TAA, TAG); however, the stop colon in *cox1*, *cox2* and *nad5* terminated in a single T, which have also been previously reported for many stoneflies like *Leuctra* sp., *Nemoura nankinensis*, *Taeniopteryx ugola* and *Doddsia occidentalis*^9,10,12^. Such translation termination could be completed by post-transcriptional polyadenylation^9,10^. The relative synonymous codon usage (RSCU) values of TTA (*Leu*), TCT (*Ser*), and CCT (*Pro*) were relatively high, whereas TCG (*Ser*) and ACG (*Thr*) were used less frequently than other codons (Fig. 2, Table 3). Most species of stoneflies show a high RSCU value of leucine while the usage of other amino acids are diverse from each other^8–12^.

**Table 3:** Codons and relative synonymous codon usage (RSCU) of protein-coding genes (PCGs) in *O. nigribasis*.

Codon	Count	RSCU	Codon	Count	RSCU	Codon	Count	RSCU	Codon	Count	RSCU
UUU(F)	248	1.55	UCU(S)	99	2.34	UAU(Y)	110	1.45	UGU(C)	39	1.77
UUC(F)	72	0.45	UCC(S)	31	0.73	UAC(Y)	42	0.55	UGC(C)	5	0.23
UUA(L)	334	3.21	UCA(S)	77	1.82	UAA(*)	0	0.00	UGA(W)	101	1.77
UUG(L)	82	0.79	UCG(S)	4	0.09	UAG(*)	0	0.00	UGG(W)	13	0.23
CUU(L)	96	0.92	CCU(P)	76	2.05	CAU(H)	54	1.32	CGU(R)	22	1.42
CUC(L)	29	0.28	CCC(P)	30	0.81	CAC(H)	28	0.68	CGC(R)	5	0.32
CUA(L)	70	0.67	CCA(P)	38	1.03	CAA(Q)	73	1.74	CGA(R)	29	1.87
CUG(L)	13	0.13	CCG(P)	4	0.11	CAG(Q)	11	0.26	CGG(R)	6	0.39
AUU(I)	249	1.72	ACU(T)	93	1.75	AAU(N)	112	1.49	AGU(S)	36	0.85
AUC(I)	41	0.28	ACC(T)	41	0.77	AAC(N)	38	0.51	AGC(S)	12	0.28
AUA(M)	167	1.59	ACA(T)	74	1.39	AAA(K)	53	1.41	AGA(S)	80	1.89
AUG(M)	43	0.41	ACG(T)	5	0.09	AAG(K)	22	0.59	AGG(S)	0	0.00
GUU(V)	104	1.77	GCU(A)	94	1.79	GAU(D)	57	1.56	GGU(G)	83	1.37
GUC(V)	17	0.29	GCC(A)	42	0.80	GAC(D)	16	0.44	GGC(G)	15	0.25
GUA(V)	92	1.57	GCA(A)	61	1.16	GAA(E)	59	1.53	GGA(G)	106	1.75
GUG(V)	22	0.37	GCG(A)	13	0.25	GAG(E)	18	0.47	GGG(G)	38	0.63

### Transfer RNA genes

**Figure 3:**
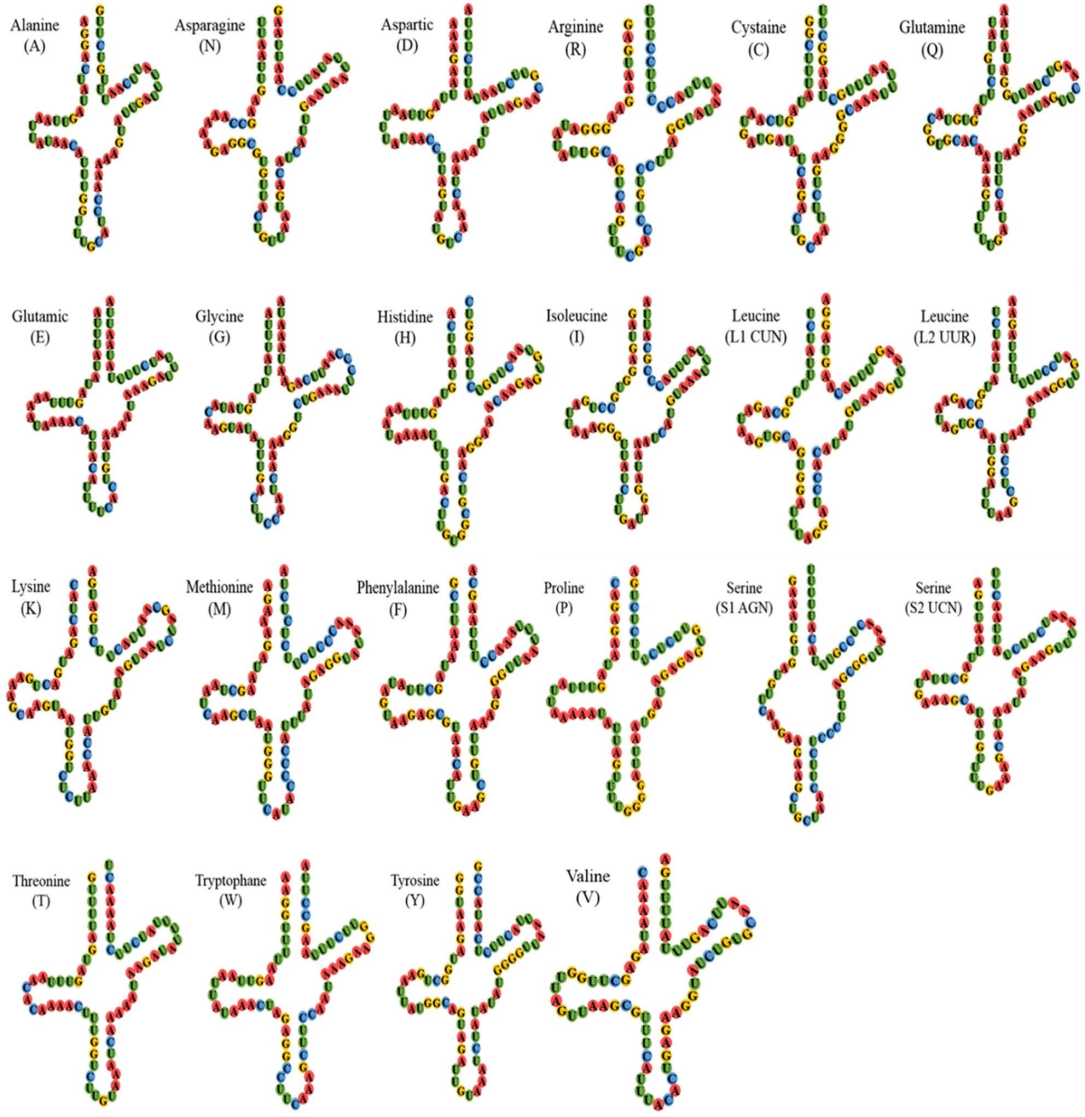
Predicted secondary structures of tRNAs from *O. nigribasis*. tRNAs are labelled with the abbreviations of their corresponding amino acids.

The typical set of 22 tRNA genes was observed in the *O. nigribasis* mitogenome, and the combined length and mean A + T content was 1426 bp and 70.2%, respectively (Table 2). Fourteen tRNAs were encoded in a clockwise orientation, whereas the remaining eight were transcribed counterclockwise (Fig. 1, Table 1). Apart from *trnSer* (*AGN*), where the dihydrouridine (DHU) arm was absent, and that is a very common feature of mitochondrial tRNA-Ser conserved in mammals and some insects^9–12,25^, the other 21 tRNAs exhibited the representative cloverleaf secondary structure (Fig. 3) that is typical of other metazoan mitogenomes. The tRNAs contained some mismatched base pairs, and many of these contained G-U pairs (Fig. 3).

### Ribosomal RNA genes

Two rRNA genes were predicted from the *O. nigribasis* mitogenome and the combined length and A + T content was 2181 bp and 71.5%, respectively (Table 2). The two rRNA genes (*rrnL*, *rrnS*) generally map between *trnLeu* (*CUN*) and the CR, and this location was conserved in the mtDNA of *O. nigribasis* (Fig. 1). The full-length, intact *rrnL* was 1362 bp with an A + T content of 72.8%, whereas the 819 bp *rrnS* was truncated and had a 70.2% A + T content (Table 1).

### The non-coding control region

Mitogenome control regions are highly variable and exhibit variable lengths and nucleotide composition. The *O. nigribasis* CR was slightly larger than the CR in Plecopteran insects, while the A + T content was typical of other sequenced species. The *O. nigribasis* CR mapped between *rrnS* and *trnIle*, a location that is relatively conserved among stoneflies (Fig. 1).

**Figure 4:**
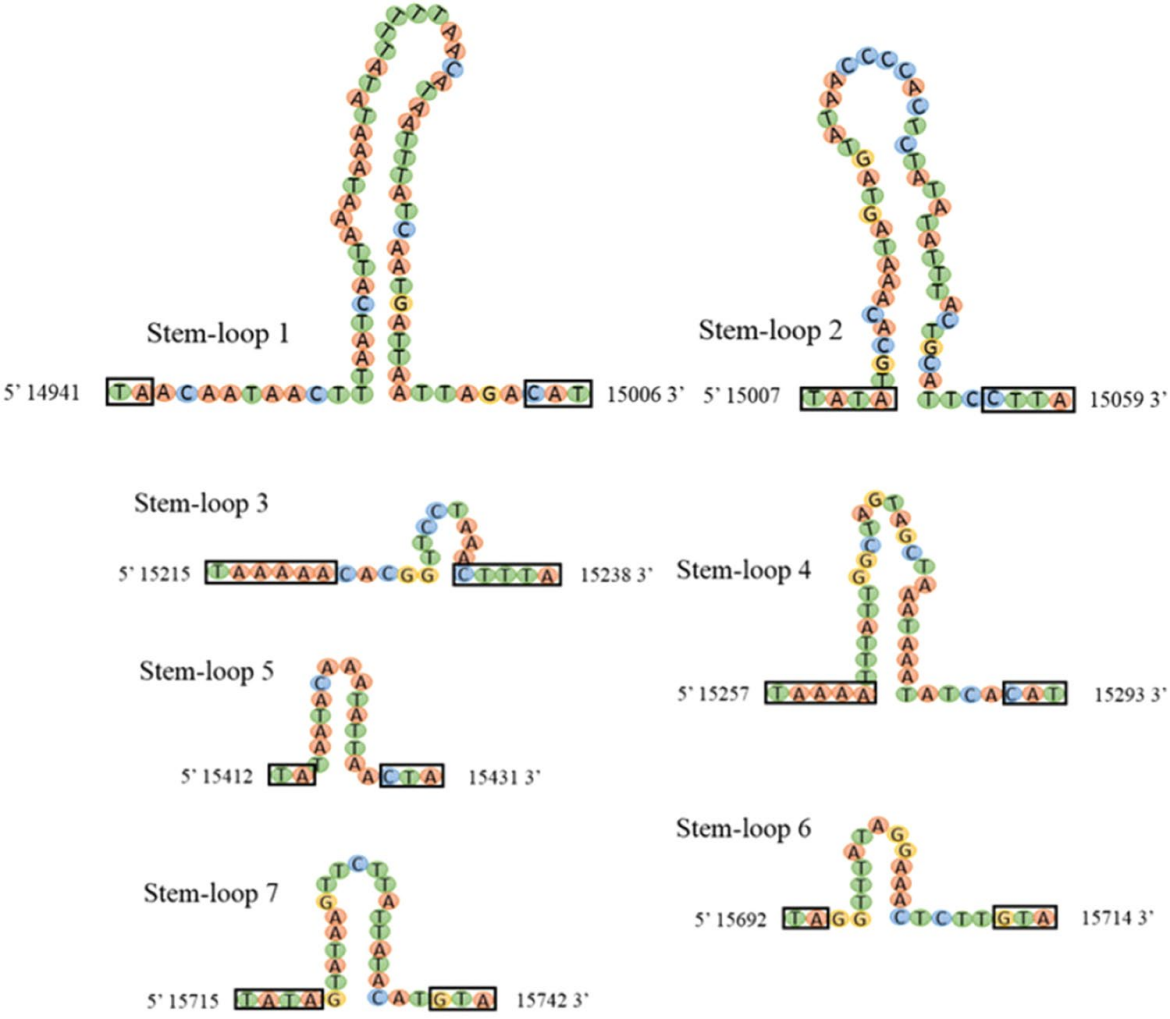
Potential stem-loop structures in the control region of *O. nigribasis*. The bilateral nucleotide motifs of each stem-loop structure [(TA)_n_, CAT, T(A)_n_, C(T)_n_A, GTA] are bounded by black rectangles.

Seven stem-loop (SL) structures mapped in the CR at the following positions: 14,941–15,006 bp; 15,007–15,059 bp; 15,215–15,238 bp; 15,257–15,293 bp; 15,412–15,431 bp; 15,692–15,714 bp; and 15,715–15,742 bp (Fig. 4). Four tandem repeats mapped between 15,525–15,661 bp. The remaining sequences in the CR were A + T rich (Fig. 4).

### Phylogenetic analyses

The concatenated sequences of 13 PCGs from 38 additional Plecopteran species were downloaded from GenBank. The mtDNAs from two Ephemeroptera species, *Parafronurus youi* and *Isonychia ignota*, served as outgroups (Table 5) because they are relatively close to stonefly in classification. ClustalX was used to align the amino acid sequences of the 13 PCGs, and MrBayes v. 3.1.2 and IQ-Tree v. 1.6.12 were utilized to generate the topology by Bayesian inference (BI) and maximum likelihood (ML) analysis, respectively.

**Figure 5:**
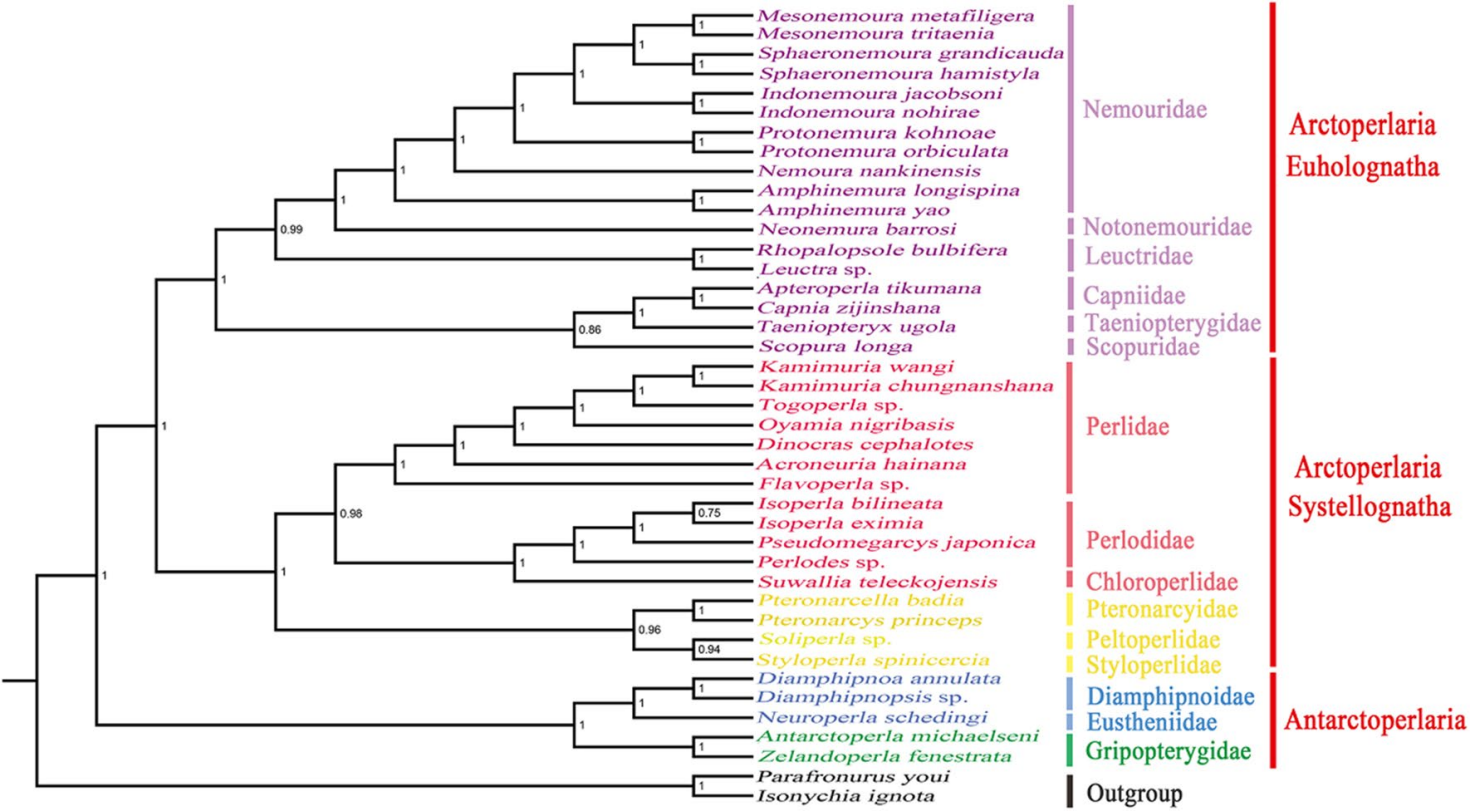
Phylogenetic relationships among 39 stoneflies based on Bayesian inference (BI). Numbers at the nodes represent posterior probabilities. Family and infraorder names are marked to the right of each species. *Parafronurus youi* and *Isonychia ignota* served as outgroup species.

**Figure 6:**
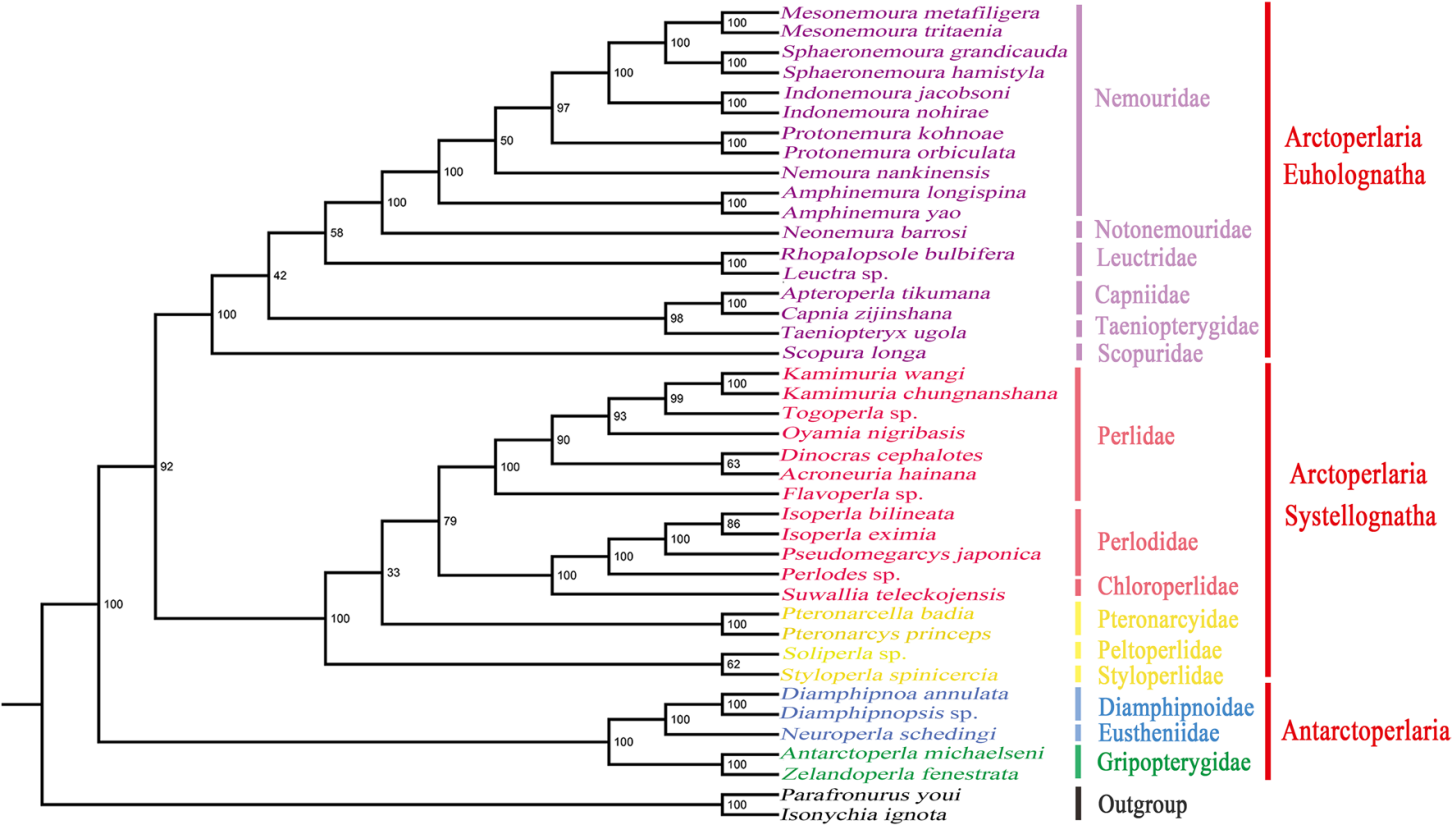
Phylogenetic relationships among 39 stoneflies based on maximum likelihood (ML) analysis. Numbers at the nodes represent bootstrap values. Family and infraorder names are marked to the right of each species. *Parafronurus youi* and *Isonychia ignota* served as outgroup species.

The two trees showed a high degree of similarity (Fig. 5, 6), excluding individual species such as the ones in Scopuridae and Pteronarcyidae. However, it is important to note that the nodal support values of the BI tree (Fig. 5) were more credible based on a previous analysis by ML^11^. Sequence data of selected southern hemisphere families were analyzed; the suborder Antarctoperlaria, including Gripopterygidae, Diamphipnoidae and Eustheniidae, was separated from other stonefly families that affiliated with Arctoperlaria, which is distributed in the northern hemisphere. This finding differs from the conclusion that Gripopterygidae could not be separated from other Arctoperlarian families in Shen & Du, 2019^12^, while consistent with Ding, 2019^23^. The two clades of Arctoperlaria, Euholognatha and Systellognatha, were strongly supported at the family level as monophyletic clades. In the infraorder Euholognatha, it was explicit that Leuctridae was clustered with the group of Nemouridae + Notonemouridae and Taeniopterygidae was recovered as the sister group of Capniidae. However, the phylogenetic relationship of Scopuridae, which need more datasets and independent evidence, was difficult to determine. Scopuridae was close to Taeniopterygidae and Capniidae based on BI analysis but clustered with other Euholognatha in the ML tree. From the perspective of Systellognatha, the monophyletic relationships in the superfamily Perloideae could be highly advocated as (Perlidae + (Perlodidae + Chloroperlidae)), even though marginal divergence has been reported^11,16^. However, the phylogenetic relationship within the superfamily Pteronarcyoidea is more controversial. As shown in the BI tree (Fig. 5), Styloperlidae was more closely related to Peltoperlidae and clustered with Pteronarcyidae, which was consistent with morphology but inconsistent with phylogeny of ((Pteronarcyidae + Styloperlidae) + Peltoperlidae)^11^. Pteronarcyidae was included in the clade containing Perloideae and clustered with Styloperlidae and Peltoperlidae in the ML tree. Similar discrepancies have been reported in related studies^23^ and are potentially caused by the use of different algorithms and models.

Increasing numbers of stonefly mtDNAs are undergoing sequence analysis. Thus, it is likely that controversial phylogenetic relationships will eventually be resolved and the phylogeny of Plecoptera can be more accurately presented based on increased numbers of mitogenomes. It is worth looking forward to that more genes just like nuclear genes can also help to improve the phylogeny of stoneflies.

## Methods

### Sample preparation and mitogenome amplification

This study was conducted without harming protected or endangered species, and all research activities were authorized. Specimens of *O. nigribasis* were collected from Benxi (Liaoning Province, China; July, 2018) and preserved in 100% ethanol. DNA extraction was performed using instructions supplied with the Column mtDNAout kit (Tianda Beijing, China). Universal or specifically- designed primers were used to amplify mitochondrial genes in long overlapping fragments (Table 4). LA-PCR and consecutive specific PCR amplifications were conducted using conditions described previously^10^. PCR products were purified with the Axygen DNA Gel Extraction Kit (Axygen Biotechnology, Hangzhou, China), separated in 1.0% agarose gels, and sequenced by Map Biotech Co. (Shanghai, China).

**Table 4:** Primers for PCR amplification and sequence analysis.

Name	Primers sequences (5′-3′)
ON003	F: TAAAATTAAATCCTTAGAATAAAATCCTG
	R: GAATTTTATTAGGTTGAGATGGTTTAG
ON005	F: AGGTTGAACTGTTTATCCCCCTCTC
	R: GAATTTTATTAGGTTGAGATGGTTTAG
ON007	F: CTTTCCACCCTTACTTTTCATTT
	R: TACCTTAGGGATAACAGCGTAAT
ON014	F: AACAACTAAAACCCCAATAACTCTT
	R: CAATAAAAGGGAGTACAAAATGG
ON020	F: ACCCCAATAAAATATGAATAACTATG
	R: GTTCAACCTGTTCCTGCTCCGTTT
ON-16S	F: CGCCTGTTTATCAAAAACAT
	R: CCGGTCTGAACTCAGATCACGT
ON-COI	F: GCCCACGCCTTYGTAATAATTTTCT
	R: GCAACTGCTCAAACAAATAAAGG

### Mitogenome assembly and annotation

Mitogenome assembly was conducted with CodonCode Aligner (http://www.codoncode.com/aligner/). Genes encoding PCGs and rRNAs were identified using mtDNA sequences of other Plecoptera and boundaries were defined with ORF finder (https://www.ncbi.nlm.nih.gov/orffinder/). CGView Server^26^ and MITOS^27^ were used to draft mtDNA maps and predict tRNA secondary structure; nucleotide composition was obtained with MEGA v. 6.0^28^. Formulas for AT-skew [A – T]/[A + T] and GC-skew [G – C]/[G + C]^29^ were used to derive AT and GC composition, respectively. Tandem Repeats Finder (http://tandem.bu.edu/trf/trf.advanced.submit.html) and DNAMAN v. 6.0.3 was utilized to detect tandem repeats in the putative CR and to predict stem-loop (SL) structures, respectively. The mtDNA sequence of *O. nigribasis* was deposited in GenBank as accession no. MN548290.

### Phylogenetic analysis

The phylogeny of 39 Plecoptera mitogenomes were analyzed, including 18 Euholognathas, 16 Systellognathans, 5 Antarctoperlarias. *Parafronurus youi* and *Isonychia ignota* from the family Ephemeroptera were used as outgroup species (Table 5). Thirteen PCGs were ordered and assembled using MAFFT^30^ and SequenceMatrix v. 1.7.8 ^31^, and stop codons were excluded. Nucleotide saturation was detected using DAMBE v. 5.2 prior to constructing phylogenetic trees; optimal displacement models (GTR + G + I) were deduced using PartitionFinder v. 2.1.1 ^32^ with Bayesian Information Criterion (BIC) and a greedy search algorithm with unlinked branch lengths. Bayesian inference analyses were conducted with MrBayes v. 3.1.2 (http://morphbank.ebc.uu.SE/mrbayes/) and 20 million generations; sampling occurred every 100 generations with four chains (three hot and one cold), and a burn-in of 25% trees^33^ Tracer v. 1.5 (http://tree.bio.ed.ac.uk/) (effective sample size > 200) was used to examine the stationarity of all runs. For maximum likelihood, 10,000 ultrafast bootstrap (UFBoot) approximations were performed with IQ-Tree v. 1.6.12 (http://www.iqtree.org/) ^34,35^. Ultimately, TreeView v. 5.1.6 or FigTree v. 1.4.2 was used to transform data into phylogenetic trees and for data annotation.

**Table 5:** Species of Plecoptera and Ephemeroptera used for phylogeny.

Order	Family	Species	GenBank accession no
Plecoptera	Perlidae	*Kamimuria wangi*	KC894944
		*Kamimuria chungnanshana*	KT186102
		*Togoperla* sp.	KM409708
		*Dinocras cephalotes*	KF484757
		*Acroneuria hainana*	KM199685
		*Flavoperla* sp.	MK905206
		*Oyamia nigribasis*	MN548290
	Perlodidae	*Isoperla bilineata*	MF716959
		*Isoperla eximia*	MG910457
		*Perlodes* sp.	MF197377
		*Pseudomegarcys japonica*	MG910458
	Chloroperlidae	*Suwallia teleckojensis*	MF198253
	Pteronarcyidae	*Pteronarcys princeps*	AY687866
		*Pteronarcella badia*	KU182360
	Styloperlidae	*Styloperla spinicercia*	KX845569
	Peltoperlidae	*Soliperla* sp.	MF716958
	Capniidae	*Apteroperla tikumana*	KR604721
		*Capnia zijinshana*	KX094942
	Nemouridae	*Nemoura nankinensis*	KY940360
		*Amphinemura longispina*	MH085446
		*Amphinemura yao*	MH085447
		*Indonemoura jacobsoni*	MH085448
		*Indonemoura nohirae*	MH085449
		*Mesonemoura metafiligera*	MH085450
		*Mesonemoura tritaenia*	MH085451
		*Protonemura kohnoae*	MH085452
		*Protonemura orbiculata*	MH085453
		*Sphaeronemoura grandicauda*	MH085454
		*Sphaeronemoura hamistyla*	MH085455
	Leuctridae	*Rhopalopsole bulbifera*	MK111419
		*Leuctra* sp.	MK568475
	Taeniopterygidae	*Taeniopteryx ugola*	MG589786
	Notonemouridae	*Neonemura barrosi*	MK111418
	Gripopterygidae	*Zelandoperla fenestrata*	KY522907
		*Antarctoperla michaelseni*	MK111413
	Diamphipnoidae	*Diamphipnoa annulata*	MK111416
		*Diamphipnopsis* sp.	MK111417
	Eustheniidae	*Neuroperla schedingi*	MK111415
	Scopuridae	*Scopura longa*	MH510071
Ephemeroptera	Heptageniidae	*Parafronurus youi*	EU349015
	Isonychiidae	*Isonychia ignota*	HM143892
